# Serum levels of inflammatory and regulatory cytokines in patients with hemorrhagic fever with renal syndrome

**DOI:** 10.1186/1471-2334-11-142

**Published:** 2011-05-23

**Authors:** Ana Saksida, Branka Wraber, Tatjana Avšič-Županc

**Affiliations:** 1 Institute of Microbiology and Immunology, Faculty of Medicine, University of Ljubljana, Ljubljana, Slovenia

## Abstract

**Background:**

Hantaviruses are the causative agents of two zoonotic diseases: hemorrhagic fever with renal syndrome (HFRS) and hantavirus cardiopulmonary syndrome (HCPS). The pathogenesis of HFRS is poorly understood. However, it has been suggested that immune mechanisms, including cytokines, might have an important role in HFRS pathogenesis. Thus, the aim of our study was to investigate cytokine profiles in serum samples of HFRS patients from Slovenia and explore a possible correlation between cytokine levels and disease severity.

**Methods:**

Acute-phase serum samples from 52 patients, diagnosed with DOBV infection, and 61 patients, diagnosed with PUUV infection, were included in this study. Patients were divided into two groups - severe or mild - based on disease severity. Levels of IL-10, IL-12, INF-γ and TNF-α were measured in the serum samples with commercial ELISA tests.

**Results:**

Increased levels of IL-10, INF-γ, and TNF-α were found in almost all the serum samples tested. On average, higher concentrations were detected in patients infected with DOBV than PUUV. Furthermore, significantly higher levels of IL-10 (*P *= 0.001) and TNF-α (*P *= 0.003) were found in patients with a more severe clinical course of disease. The same association between IL-10 (*P *< 0.001) and TNF-α (*P *= 0.021), and the severity of the disease was observed also when only patients infected with DOBV were considered. No differences in cytokine concentrations according to disease severity were observed in patients infected with PUUV. Concentrations of serum IL-12 in HFRS patients were in the normal range, however, higher levels were detected in patients infected with PUUV than in patients infected with DOBV.

**Conclusions:**

We suggest that imbalance in production of proinflammatory and regulatory cytokines might be in part responsible for a more severe course of HFRS.

## Background

Hantaviruses, rodent-borne bunyaviruses, are the etiologic agents of two zoonotic diseases: hemorrhagic fever with renal syndrome (HFRS) and hantavirus cardiopulmonary syndrome (HCPS) [1].

In HFRS, the severity of the disease varies depending on the particular virus involved. Hantaan (HTNV) and Dobrava viruses (DOBV) tend to produce the most severe disease, with mortality rates 5-10%. Puumala virus (PUUV) usually causes a less severe disease, called nephropathia epidemica (NE), with mortality rate of less than 1% and Seoul virus (SEOV) typically produces disease of intermediate severity with a 1% mortality rate. Clinically, HFRS presents with sudden onset of fever, headache and myalgia with renal impairment as the predominant organ manifestation. Clinical symptoms also include thrombocytopenia and, in severe cases, hemorrhages as a result of the vascular endothelium disfunction [2-4].

The pathogenesis of HFRS, like that of many other viral hemorrhagic fevers, is poorly understood. Because of the lack of suitable animal models, pathogenesis research is limited to in vitro and rare clinical studies. Endothelial cells and monocytes are thought to be the primary cell targets of the viruses, but infection doesn't seem to have any direct cytopathic effect on these cells. Therefore, it has been suggested that HFRS pathogenesis is likely to be a complex multifactorial process that includes contributions from immune responses, platelet dysfunction, disregulation of endothelial cell barrier functions and hosts' genetic factors [5-7]. Among immune parameters, certain cytokines such as IL-1, IL-6, IL-10 and TNF-α were suggested to be involved in the pathogenesis, since increased levels of these cytokines were found in patients with HFRS [8-11].

The presence of HFRS in Slovenia was first reported in 1954. Since then, over 300 cases occurring sporadically or in small epidemics have been documented. Both severe and mild clinical courses of the disease are observed, with an overall mortality rate of 3,3%. It has been demonstrated earlier that in Slovenia DOBV and PUUV co-exist in a single endemic region and are capable of causing HFRS with significant differences in disease severity as well as mortality. Namely, all fatal HFRS cases so far have been caused by DOBV infection, resulting in 8,3% mortality rate for DOBV associated HFRS. Furthermore, differences in disease severity within the HFRS cases caused by DOBV have been noticed. [[12,13], unpublished data].

In light of previous findings, the aim of our study was to investigate cytokine profiles in serum samples of HFRS patients from Slovenia. To the best of our knowledge, this is the first study describing serum cytokine levels in patients infected with DOBV. In addition, comparison of the serum levels of cytokines in patients infected with DOBV and PUUV, causative agents of HFRS, is described for the first time. We also explore a possible correlation between cytokine levels and disease severity.

## Methods

### Study subjects and sample collection

In Slovenia, 298 HFRS cases were reported between 1985 and July 2010. One-hundred-and-twelve (37.6%) of the patients were diagnosed with DOBV and 186 (62.4%) with PUUV infection. Acute-phase serum samples from 52 patients, diagnosed with DOBV infection, and 61 patients, diagnosed with PUUV infection, were included in this study. The patients were treated at several infectious disease hospitals across the country and clinical data for the patients was collected retrospectively. During the course of the disease, the clinical diagnosis was confirmed serologically by an indirect immunofluorescence assay (IFA) and enzyme-linked immuno assay (ELISA) IgM and IgG tests with HTNV, PUUV and DOBV antigens as described previously [12]. The remains of the samples were stored in our laboratory at -80°C.

Patients, infected with DOBV or PUUV, were divided into two groups - severe or mild-moderate - based on disease severity. Categorization was based on clinical and laboratory parameters employed in the proposed Croatian scale for grading the disease severity in patients with HFRS (Table 1) [14]. Namely, patients with a total score of 12 or more were defined as having a severe course of the disease.

**Table 1:** Scale for grading the disease severity in patients with HFRS

Signs and symptoms		Score
1. SHOCK, HYPOTENSION ^a^		
Shock		10
Hypotension, systolic blood pressure < 90 mm Hg		5
Tachycardia, heart rate > 120/min		2

2. HAEMORRHAGES ^a^		
Massive: blood transfusion needed		5
Internal (melena, hematemesis, hemoptysis, intracranial)		3
Epistaxis		2
Petechiae, conjuctival injection, enanthema		1

3. GENERAL SYMPTOMS		
Body temperature > 40°C		2
Seizure		2
Blurred vision		1
Vomiting and diarrhea		1
Anuria/oliguria		1
Dialysis requirement		1

4. Laboratory parameters		
Serum urea and/or creatinine	> 5-times the normal value	5
	> 4-times the normal value	4
	> 3-times the normal value	3
	> 2-times the normal value	2
Trombociti	< 20 × 10^9^/L	2
	< 50 × 10^9^/L	1
AST, ALT	> 5-times the normal value	2
	3-4-times the normal value	1
Lung X-ray	pneumonitis	2
	pleural effusion	1

^a ^In group 1 and 2, only the signs/symptoms with the highest score are considered

Since the study was retrospective, informed consent from the patients was not obtained. Instead, the research was approved by the National Medical Ethics Committee of the Republic of Slovenia. Also, the principles of the Helsinki Declaration, the Oviedo Convention on Human Rights and Biomedicine and the Slovene Code of Medical Deontology were followed in the conduct of this research. No additional sample was taken for the purpose of the study.

### Cytokine assays

Serum levels of IL-10, IL-12, INF-γ and TNF-α were measured retrospectively with commercial ELISA tests. Namely, for the determination of IL-10, IL-12 and INF-γ, Endogen^® ^Human IL-10 ELISA kit, Endogen^® ^Total Human IL-12 ELISA kit, and Endogen^® ^Human INF-γ ELISA kit (all Pierce Biotechnology, Inc.) were used. ELISA Quantikine HS Human TNF-α Immunoassay (R&D Systems, Inc.) test was used to measure TNF-α concentrations. The limits of detection for IL-10, IL-12, INF-γ and TNF-α were < 3 pg/ml, < 5 pg/ml, < 2 pg/ml and < 0.106 pg/ml, respectively. Normal values for IL-10, IL-12, INF-γ and TNF-α were determined to be < 7.65 pg/ml, < 258 pg/ml, < 1 pg/ml and < 2.42 pg/ml, respectively. They were measured in serum samples of healthy adult blood donors by the Laboratory for Allergy and Cytokine Diagnostics at the Institute of Microbiology and Immunology, Medical Faculty, Ljubljana, Slovenia.

### Statistical analysis

Results were analysed using the statistical software package SPSS 17.0 for Windows (SPSS Inc.). The relationship between the variables and the clinical classification was evaluated using Mann-Whitney or Kruskal-Wallis tests for continuous variables. The correlation among variables was assessed with Pearson's test and multiple linear regression. *P *values of < 0.05 were considered significant.

## Results

### Clinical data

Fifty-two patients with confirmed acute DOBV infection and 61 patients with confirmed PUUV infection were enrolled in the study. On the basis of their case records, 30 patients, infected with DOBV, were categorized as having severe disease and 22 as having mild-moderate disease. From patients infected with PUUV 19 were categorized as having severe disease and 42 as having mild-moderate disease. The main clinical characteristics and laboratory values of patients' groups are summarized in Tables 2 and 3.

**Table 2:** Occurence of different clinical and laboratory findings in patients with HFRS according to the causative agent

Finding	DOBV (n = 52)	PUUV (n = 61)
Fever > 39°C	89%	81%
Backache or abdominal pains	90%	61%
Blurred vision	40%	35%
Haemorrhagic manifestations ^a^	55%	28%
Thrombocytopenia (< 50 × 10^9^cells/l)	42%	38%
Oliguria (< 0.5 l urine/24 hrs)	66%	39%
Serum creatinine (μmol/l) ^b^	622 (89-1157)*	368 (77-1420)*
Urea (mmol/l) ^b^	30 (5,1-76,2)*	17,6 (4,2-48,2)*
Dialysis requirement	49%	9%

^a ^Most frequent haemorrhagic manifestations: conjuctival injection, petechiae, hematemesis, melena.

^b ^Normal values: thrombocytes 140-360 × 10^9^cells/l, serum creatinine 44-97 μmol/l, urea 2,8-7,5 mmol/l.

* Given as mean (min-max) of highest values detected

**Table 3:** Comparisson of different clinical and laboratory findings in patients infected with DOB Vand PUUV according to disease severity

Finding	DOBV		PUUV	
	
	mild-moderate (n = 22)	severe (n = 30)	mild-moderate (n = 42)	severe (n = 19)
Haemorrhages	36,4%	76,7%	21,4%	47,4%
Thrombocytopenia < 50 × 10^9^/l	27,3%	56,7%	33,3%	52,6%
Oliguria < 0.5 l/24 hrs	31,8%	73,3%	19,0%	73,7%
Dialysis requirement	18,2%	76,7%	0%	36,8%
Serum creatinine (μmol/l)	418 (89-1058)*	781 (311-1157)*	246 (77-742)*	669 (271-1420)*
Urea (mmol/l)	21,9 (5,1-51,6)*	36,4 (10,8-76,2)*	13,3 (4,2-48,2)*	28,1 (12,7-45,7)*

* Given as mean (min-max) of highest values detected

### Cytokine levels

**Figure 1:**
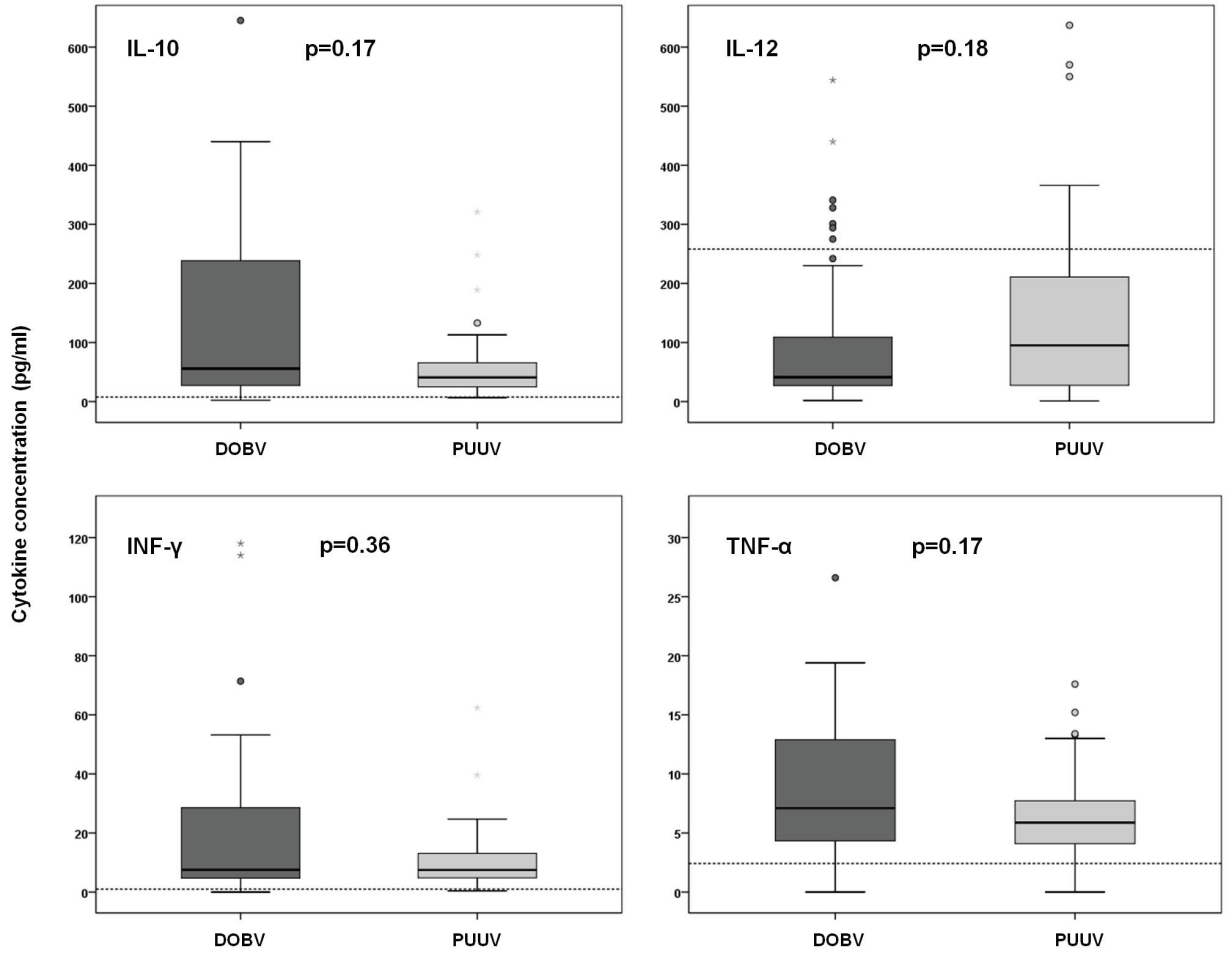
**Comparison of serum cytokine concentrations in patients with acute HFRS according to the causative agent of the disease**. Horizontal bars show median values. Dotted lines represent the upper limits of normal values.

Concentrations of IL-10, IL-12 and INF-γ were determined retrospectively in all serum samples. Due to the limited volume of available samples, levels of TNF-α could only be measured in 46 and 55 serum samples of patients infected with DOBV and PUUV, respectively (Table 4). Elevated levels of IL-10, INF-γ and TNF-α were detected in almost all the samples tested, regardless of the causative agent or the clinical course of the disease. On the contrary, concentrations of IL-12 were in the normal range, even more, detected levels of IL-12 in patients infected with DOBV were significantly lower than values detected in healthy blood donor volunteers (*P *= 0.006) (data not shown). Serum concentrations of IL-12 were higher in patients infected with PUUV than in patients infected with DOBV, but the difference was not significant (*P *= 0.18). Also serum levels of IL-12 detected in PUUV infected patients did not differ from normal values (*P *= 0.34). In DOBV infected patients mean concentrations of IL-10 and INF-γ were higher than in patients infected with PUUV, however the differences were not significant (*P *= 0.17 and *P *= 0.36). No considerable difference was observed in TNF-α levels between patients infected with DOBV and PUUV (*P *= 0.17) (Figure 1).

**Table 4:** Cytokine concentrations in acute serum samples of patients with HFRS according to the causative agent and disease severity

Patient group	Mean levels (min-max) of cytokines (pg/ml)			
	
	IL-10	IL-12	INF-γ	TNF-α
DOBV	292.5 (2.24-6600)	100.4 (1.94-544)	89.1 (0-1916)	9.8 (0-62.5)
severe	474.4 (12.4-6600)	97.7 (10.9-544)	131.5 (0-1916)	12.3 (1.87-62.5)
mild-moderate	44.4 (2.24-328)	104.0 (1.94-440)	31.4 (0-354)	6.3 (0-17)

PUUV	130.3 (6.63-2120)	139.2 (0.98-637)	24.1 (0.43-692)	10.8 (0-138)
severe	89.9 (15.5-731)	151.1 (2.49-570)	46.6 (1.61-692)	7.6 (2.47-15.2)
mild-moderate	148.6 (6.63-2120)	133.8 (0.98-637)	14.0 (0.43-203)	12.3 (0-138)

**Figure 2:**
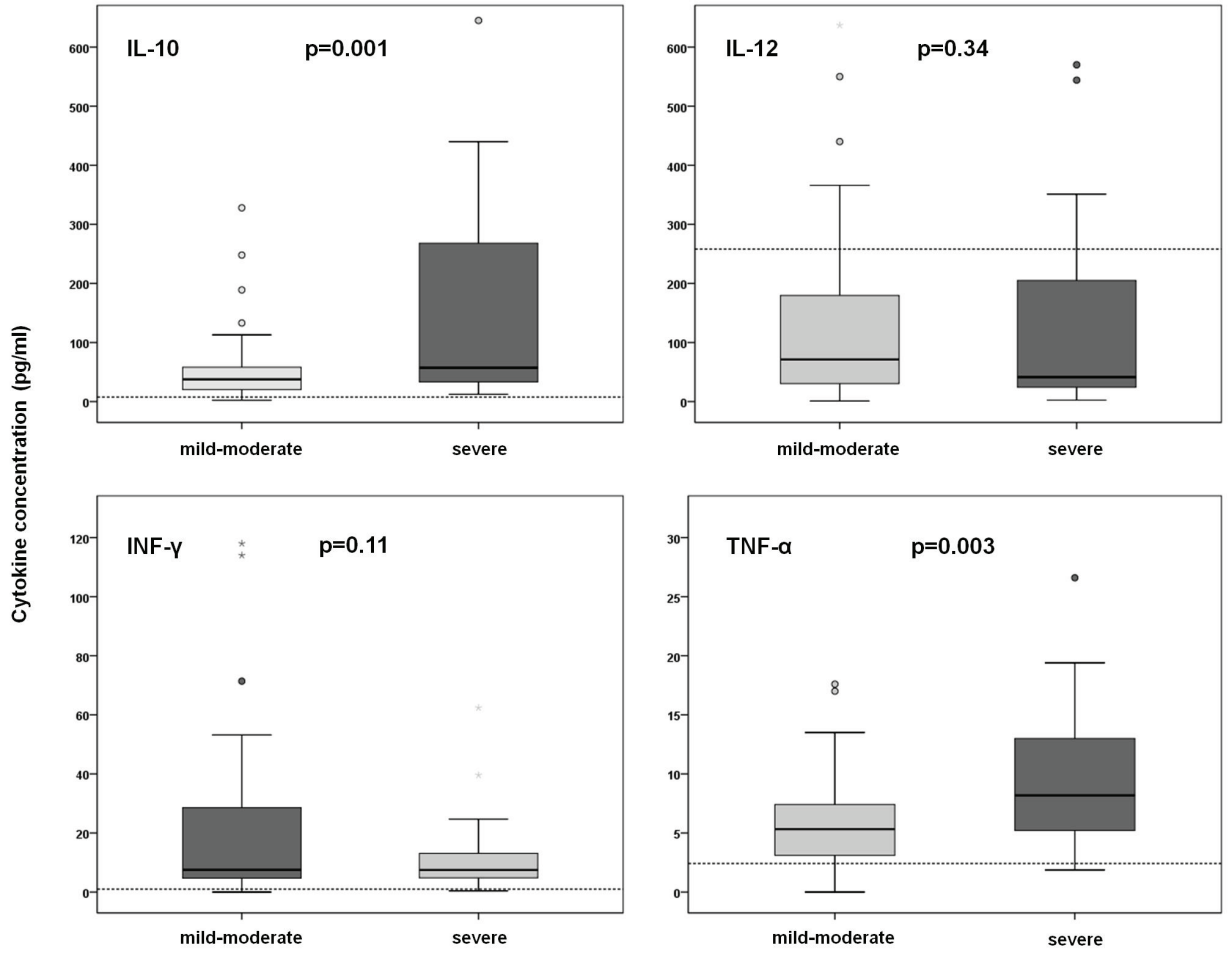
**Comparison of serum cytokine concentrations in patients with acute HFRS according to the clinical course of the disease**. Horizontal bars show median values. Dotted lines represent the upper limits of normal values.

**Figure 3:**
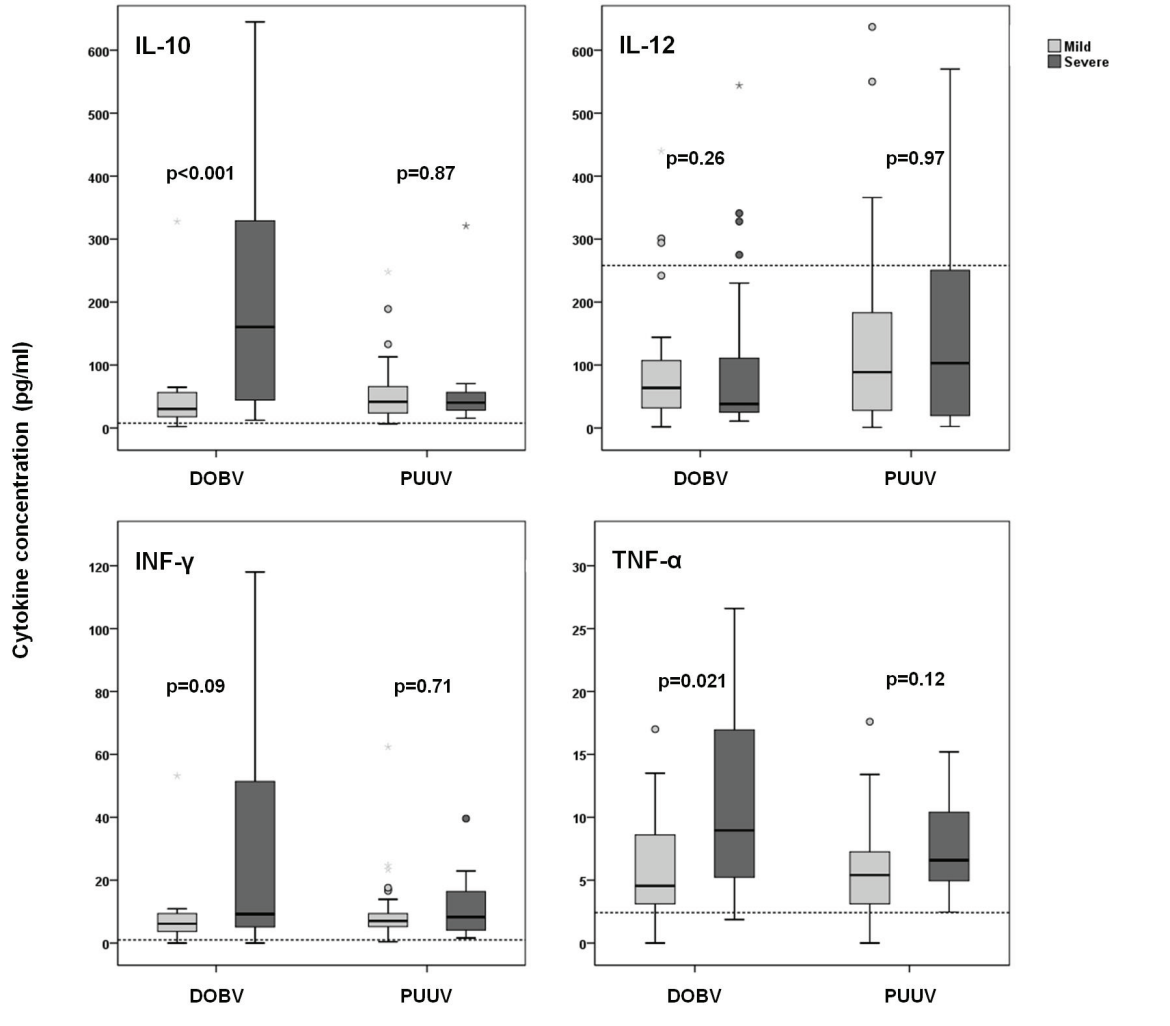
**Comparison of serum cytokine concentrations in patients with acute HFRS according to the causative agent (DOBV or PUUV) and clinical course of the disease**. Horizontal bars show median values. Dotted lines represent the upper limits of normal values.

When patients were divided according to disease severity, regardless of the causative agent, significantly higher levels of IL-10 and TNF-α were detected in the patients with severe disease course (*P *= 0.001 and *P *= 0.003). (Figure 2). In DOBV infected patients higher levels of all measured cytokines were detected in patients with severe disease course than in patients with mild-moderate disease course. Significant differences were observed for IL-10 (*P *< 0.001) and TNF-α (*P *= 0.021), while detected values of IL-12 and INF-γ were not significantly different between these two patients' groups (*P *= 0.26 and *P *= 0.09) (Figure 3).

For the patients diagnosed with PUUV infection, no significant differences in cytokine concentrations were observed regarding the disease severity. Higher mean levels of IL-12 (*P *= 0.97) and INF-γ (*P *= 0.71) were detected in patients with severe than with mild-moderate disease course, while the opposite was true for IL-10 (*P *= 0.87) and TNF-α (*P *= 0.12) (Figure 3).

When cytokine levels were compared according to the day of illness the sample was taken, no time dependence could be observed for any group of patients, regardless of the causative agent or the clinical course of the disease (data not shown).

## Discussion

Growing evidence exists that immune mechanisms rather than direct viral cytopathology are responsible for the changes in vascular permeability, the principal abnormality, in both HFRS and HCPS [6,15]. Activation of hantavirus specific CD8+ T cells and cytokine production seem to be of special importance, since high levels of these cells have been found in blood, lung, and kidney tissues of HCPS and HFRS patients [16-18].

This study is the first to describe serum cytokine levels in patients infected with DOBV and also to compare the detected levels in patients infected with DOBV and PUUV. In our study, increased levels of IL-10, INF-γ, and TNF-α were found in serum samples of most HFRS patients. On average, higher concentrations were detected in patients infected with DOBV than PUUV. Furthermore, in patients infected with DOBV, higher levels of these cytokines were found in patients with a more severe clinical course of disease. No differences in cytokine concentrations according to disease severity were observed in patients infected with PUUV. It has previously been established, that severe cases of HFRS in Slovenia are mainly caused by DOBV, while infection with PUUV usually results in a milder form of disease [12,13]. This has once again been confirmed with patients included in this study, since the occurrence and intensity of clinical and laboratory findings were higher in patients infected with DOBV than PUUV. Therefore, it is expectable, that the differences in disease severity in patients infected with PUUV are smaller.

Increased levels of TNF-α have also been demonstrated in studies of patients with Korean hemorrhagic fever and patients with HCPS. However, no association with disease severity could be established in either of these studies [8,19,20]. Increased levels of TNF-α, IL-6 and IL-10 have previously been detected in patients with NE, where TNF-α levels were found to correlate with hypotension and serum NO levels and plasma IL-6 levels were associated with a more severe form of NE [9,11]. In addition, increased IL-10 and TNF-α production was demonstrated in cynomolgous monkeys infected with wild-type PUUV, where highest concentrations of TNF-α were detected in the most affected monkey [21]. High IL-10 and TNF-α serum values were also found in patients with severe course of dengue hemorrhagic fever and Argentine hemorrhagic fever, which have similar clinical presentations to HFRS [22-24].

On the basis of the results of our and previous studies, we believe that imbalance in production of proinflammatory and regulatory cytokines might be associated with disease severity in HFRS. Similar has been proposed for HCPS, where a mixed Th1/Th2 immune response was observed in serum samples of HCPS patients and the levels of Th1 cytokines correlated with disease severity [19].

In our opinion, a strong inflammatory response to virus particles and immune complexes, mainly represented by TNF-α and INF-γ, causing changes in vascular permeability, could contribute to a more severe clinical course of HFRS. Also, high production of inflammatory cytokines in turn leads to increased production of regulatory cytokines, such as IL-10, and possibly to immunosuppression. IL-10 also promotes further antibody production, likely resulting in even higher number of immune complexes, which deposit on capillary walls and in tissues. At the same time, IL-10 inhibits cell-mediated immunity by down regulating IL-12 expression, thus interfering with phagocytosis and clearance of immune complexes. This can cause formation of new inflammation sites or even a systemic inflammatory response. Our hypothesis is further supported with low IL-12 levels found in patients infected with DOBV. We believe that higher levels of IL-12 in patients infected with PUUV help to enable the development of cell-mediated immunity, resulting in a more successful clearance of immune complexes and consequently in a milder form of disease.

Similar has been hypothesized for patients with sepsis caused by gramnegative bacteria, where a strong inflammatory response to lipopolisaharid in bacterial wall, characterized by cytokines such as TNF-α, IL-1, IL-6 and IL-12, is thought to trigger a strong regulatory response, with high levels of IL-10. This could lead into immunosuppression and consequently in the worst cases cause a multi-organ failure [25].

## Conclusions

To conclude, the results of our study favor the hypothesis that cytokine production imbalance might contribute to a more severe clinical course of HFRS. Nevertheless, further immunological studies are needed to clarify, whether this imbalance is a cause or a result of the clinical symptoms we are observing. Also, additional factors are undoubtedly involved in the pathogenesis of HFRS.

## Competing interests

The authors declare that they have no competing interests.

## Authors' contributions

AS has written the manuscript, participated in the design and coordination of the study, recruited and analysed patients' data, and performed statistical analysis. BW participated in the design of the study and determined and interpreted the cytokine levels. TAŽ participated in the design and coordination of the study and helped to recruit patients' samples and clinical data. All authors have been involved in revising the manuscript and have read and approved the final version.

## Pre-publication history

The pre-publication history for this paper can be accessed here:

http://www.biomedcentral.com/1471-2334/11/142/prepub

## References

[B1] Schmaljohn C Hjelle B Hantaviruses: a global disease problem Emerging infectious diseases 1997 3 2 95 104 10.3201/eid0302.970202 9204290 PMC2627612

[B2] Cosgriff TM Mechanisms of disease in Hantavirus infection: pathophysiology of hemorrhagic fever with renal syndrome Reviews of infectious diseases 1991 13 1 97 107 1673261 10.1093/clinids/13.1.97

[B3] Jonsson CB Figueiredo LT Vapalahti O A global perspective on hantavirus ecology, epidemiology, and disease Clinical microbiology reviews 2010 23 2 412 441 10.1128/CMR.00062-09 20375360 PMC2863364

[B4] Linderholm M Elgh F Clinical characteristics of hantavirus infections on the Eurasian continent Current topics in microbiology and immunology 2001 256 135 151 11217401 10.1007/978-3-642-56753-7_8

[B5] Mackow ER Gavrilovskaya IN Hantavirus regulation of endothelial cell functions Thrombosis and haemostasis 2009 102 6 1030 1041 19967132 10.1160/TH09-09-0640

[B6] Maes P Clement J Gavrilovskaya I Van Ranst M Hantaviruses: immunology, treatment, and prevention Viral immunology 2004 17 4 481 497 10.1089/vim.2004.17.481 15671746

[B7] Terajima M Vapalahti O Van Epps HL Vaheri A Ennis FA Immune responses to Puumala virus infection and the pathogenesis of nephropathia epidemica Microbes and infection/Institut Pasteur 2004 6 2 238 245 15049335 10.1016/j.micinf.2003.10.017

[B8] Krakauer T Leduc JW Krakauer H Serum levels of tumor necrosis factor-alpha, interleukin-1, and interleukin-6 in hemorrhagic fever with renal syndrome Viral immunology 1995 8 2 75 79 10.1089/vim.1995.8.75 8825292

[B9] Linderholm M Ahlm C Settergren B Waage A Tarnvik A Elevated plasma levels of tumor necrosis factor (TNF)-alpha, soluble TNF receptors, interleukin (IL)-6, and IL-10 in patients with hemorrhagic fever with renal syndrome The Journal of infectious diseases 1996 173 1 38 43 10.1093/infdis/173.1.38 8537680

[B10] Markotic A Gagro A Dasic G Kuzman I Lukas D Nichol S Ksiazek TG Sabioncello A Rode O Rabatic S Immune parameters in hemorrhagic fever with renal syndrome during the incubation and acute disease: case report Croatian medical journal 2002 43 5 587 590 12402402

[B11] Outinen TK Makela SM Ala-Houhala IO Huhtala HS Hurme M Paakkala AS Porsti IH Syrjanen JT Mustonen JT The severity of Puumala hantavirus induced nephropathia epidemica can be better evaluated using plasma interleukin-6 than C-reactive protein determinations BMC infectious diseases 2010 10 132 10.1186/1471-2334-10-132 20500875 PMC2885391

[B12] Avsic-Zupanc T Petrovec M Furlan P Kaps R Elgh F Lundkvist A Hemorrhagic fever with renal syndrome in the Dolenjska region of Slovenia--a 10-year survey Clin Infect Dis 1999 28 4 860 865 10.1086/515185 10825051

[B13] Pal E Strle F Avsic-Zupanc T Hemorrhagic fever with renal syndrome in the Pomurje region of Slovenia--an 18-year survey Wiener klinische Wochenschrift 2005 117 11-12 398 405 10.1007/s00508-005-0359-2 16053195

[B14] Kuzman I Puljiz I Turcinov D Markotic A Turkovic B Aleraj B Andric Z Petkovic D Tutek V Herendic B The biggest epidemic of hemorrhagic fever with renal syndrome in Croatia Acta Med Croatica 2003 57 5 337 346 15011458

[B15] Khaiboullina SF Morzunov SP St Jeor SC Hantaviruses: molecular biology, evolution and pathogenesis Current molecular medicine 2005 5 8 773 790 10.2174/156652405774962317 16375712

[B16] Mori M Rothman AL Kurane I Montoya JM Nolte KB Norman JE Waite DC Koster FT Ennis FA High levels of cytokine-producing cells in the lung tissues of patients with fatal hantavirus pulmonary syndrome The Journal of infectious diseases 1999 179 2 295 302 10.1086/314597 9878011

[B17] Temonen M Mustonen J Helin H Pasternack A Vaheri A Holthofer H Cytokines, adhesion molecules, and cellular infiltration in nephropathia epidemica kidneys: an immunohistochemical study Clinical immunology and immunopathology 1996 78 1 47 55 10.1006/clin.1996.0007 8599883

[B18] Wang M Wang J Zhu Y Xu Z Yang K Yang A Jin B Cellular immune response to Hantaan virus nucleocapsid protein in the acute phase of hemorrhagic fever with renal syndrome: correlation with disease severity The Journal of infectious diseases 2009 199 2 188 195 10.1086/595834 19072554

[B19] Borges AA Campos GM Moreli ML Moro Souza RL Saggioro FP Figueiredo GG Livonesi MC Moraes Figueiredo LT Role of mixed Th1 and Th2 serum cytokines on pathogenesis and prognosis of hantavirus pulmonary syndrome Microbes and infection/Institut Pasteur 2008 10 10-11 1150 1157 18606242 10.1016/j.micinf.2008.06.006

[B20] Yang CW Bang BK Changes in serum levels of tumor necrosis factor-alpha in patients with hemorrhagic fever with renal syndrome Journal of Catholic Medical College 1992 45 819 830

[B21] Klingstrom J Plyusnin A Vaheri A Lundkvist A Wild-type Puumala hantavirus infection induces cytokines, C-reactive protein, creatinine, and nitric oxide in cynomolgus macaques Journal of virology 2002 76 1 444 449 10.1128/JVI.76.1.444-449.2002 11739712 PMC135710

[B22] Green S Vaughn DW Kalayanarooj S Nimmannitya S Suntayakorn S Nisalak A Lew R Innis BL Kurane I Rothman AL Early immune activation in acute dengue illness is related to development of plasma leakage and disease severity The Journal of infectious diseases 1999 179 4 755 762 10.1086/314680 10068569

[B23] Green S Vaughn DW Kalayanarooj S Nimmannitya S Suntayakorn S Nisalak A Rothman AL Ennis FA Elevated plasma interleukin-10 levels in acute dengue correlate with disease severity Journal of medical virology 1999 59 3 329 334 10.1002/(SICI)1096-9071(199911)59:3<329::AID-JMV12>3.0.CO;2-G 10502265

[B24] Marta RF Montero VS Hack CE Sturk A Maiztegui JI Molinas FC Proinflammatory cytokines and elastase-alpha-1-antitrypsin in Argentine hemorrhagic fever The American journal of tropical medicine and hygiene 1999 60 1 85 89 9988328 10.4269/ajtmh.1999.60.85

[B25] Cohen J The immunopathogenesis of sepsis Nature 2002 420 6917 885 891 10.1038/nature01326 12490963

